# Beyond Spheres: Evaluating Gold Nano-Flowers and Gold Nano-Stars for Enhanced Aflatoxin B1 Detection in Lateral Flow Immunoassays

**DOI:** 10.3390/bios15080495

**Published:** 2025-08-01

**Authors:** Vinayak Sharma, Bilal Javed, Hugh J. Byrne, Furong Tian

**Affiliations:** 1 School of Food Science and Environmental Health, College of Sciences and Health Technological University Dublin, D07 H6K8 Dublin, Ireland; bilal.javed@tudublin.ie; 2 Nanolab Research Centre, Physical to Life Sciences Research Hub, Technological University Dublin, D08 CKP1 Dublin, Ireland; hugh.byrne@tudublin.ie

**Keywords:** lateral flow immunoassay, aflatoxin B1, gold nano-labels, rapid analysis, point-of-care diagnostics

## Abstract

The lateral flow immunoassay (LFIA) is a widely utilized, rapid diagnostic technique characterized by its short analysis duration, cost efficiency, visual result interpretation, portability and suitability for point-of-care applications. However, conventional LFIAs have limited sensitivity, a challenge that can be overcome by the introduction of gold nanoparticles, which provide enhanced sensitivity and selectivity (compared, for example, to latex beads or carbon nanoparticles) for the detection of target analytes, due to their optical properties, chemical stability and ease of functionalization. In this work, gold nanoparticle-based LFIAs are developed for the detection of aflatoxin B1, and the relative performance of different morphology particles is evaluated. LFIA using gold nano-labels allowed for aflatoxin B1 detection over a range of 0.01 ng/mL–100 ng/mL. Compared to spherical gold nanoparticles and gold nano-flowers, star-shaped gold nanoparticles show increased antibody binding efficiency of 86% due to their greater surface area. Gold nano-stars demonstrated the highest sensitivity, achieving a limit of detection of 0.01ng/mL, surpassing the performance of both spherical gold nanoparticles and gold nano-flowers. The use of star-shaped particles as nano-labels has demonstrated a five-fold improvement in sensitivity, underscoring the potential of integrating diverse nanostructures into LFIA for significantly improving analyte detection. Moreover, the robustness and feasibility of gold nano-stars employed as labels in LFIA was assessed in detecting aflatoxin B1 in a wheat matrix. Improved sensitivity with gold nano-stars holds promise for applications in food safety monitoring, public health diagnostics and rapid point-of-care diagnostics. This work opens the pathway for further development of LFIA utilizing novel nanostructures to achieve unparallel precision in diagnostics and sensing.

## 1. Introduction

The increasing demand for the rapid detection of diverse biologically active compounds has driven the development of improved, more sensitive analytical methods. In this context, the lateral flow immunoassay (LFIA), a widely utilized point-of-care analytical method, has several benefits, including rapid analysis, prolonged stability of the test strips, user-friendliness and cost efficiency [1]. These paper-based biosensors not only offer such advantages but facilitate on-site detection, generating interest among researchers [2]. These attributes have facilitated the broadening of their applications across various sectors like food, medicine, environment surveillance and quality control [3]. The lateral flow assay is conducted using membrane-based strips, formed by different components including nitrocellulose membrane, conjugate pad, absorbent pad and sample pad. The strip components are arranged sequentially: sample pad, which receives and conditions the sample; the conjugate pad containing dried detection molecules (e.g., antibodies) conjugated with detectable labels like gold nanoparticles; the nitrocellulose membrane, where the test line and control line are immobilized to test the presence or absence of target analyte; and the absorbent pad, which wicks the sample liquid and helps to maintain the flow across the strip. The immunoreagents are pre-immobilized on the nitrocellulose membrane, which becomes activated upon the introduction of liquid samples as they travel through capillary action to the test line and control line reagents. Although the LFIA format offers several benefits, it is limited in sensitivity and quantitative accuracy for certain rapid tests [4]. Several methodologies have been employed to enhance the sensitivity of LFIA detection [4]. As per the testing process and principle, the sensitivity can be enhanced by methods including the introduction of novel nano-labels that produce readily detectable signals, optimizing test procedures and the implementation of appropriate readout techniques [5,6]. Among these methods, preparing suitable labeling material is a simple and effective approach to enhance the sensitivity [7,8].

The optical and plasmonic properties of gold nanoparticles and their biocompatibility make them an ideal candidate for their employment in lateral flow immunoassays [9]. Owing to the simplicity of their synthesis and an intense color that can be detected by the naked eye on the test strip, colloidal gold nanoparticles are widely used as labels [10]. Nanoparticles have higher surface-to-volume ratios than their bulk counterparts, which enables higher antibody binding efficiency, leading to increased sensitivity. The optical intensity of gold nanoparticles primarily depends upon their plasmonic characteristics, specifically their localized surface plasmon resonance (LSPR) band and extinction coefficient. These properties are influenced by factors such as size, shape, elemental composition and surface modifications [11]. The sensitivity of the lateral flow immunoassay is primarily determined by the affinity of the antibody used, the signal intensity of the detection label (e.g., gold nanoparticles), the quality of membrane and reagent formulation. Optimized flow dynamics and efficient antigen–antibody interactions further enhance the assay sensitivity. The sensitivity of the assay increases with increase in size of the spherical AuNPs and is limited only by diffusion of gold nanoparticles through the nitrocellulose membrane. Larger-size gold nanoparticles increase the sensitivity of LFIA, but they are unstable and require higher antibody concentration to generate a labeled and stable reagent [12]. Gold nanoparticles, including nano-rods [13], nano-flowers [14] and gold nano-stars [15], exhibit greater chemical stability, due to their more complex three-dimensional configurations [16]. Many studies have demonstrated surfactant-free methods to generate high-yield monodispersed gold nano-stars [17] and gold nano-flowers [18], which are stable and can be employed as labels in lateral flow immunoassay.

The majority of the systematic studies have been performed using conventional spherical gold nanoparticles (AuNPs) in lateral flow immunoassays for the detection [19,20]. Among all sizes, 30–40 nm have been reported to be optimal, since smaller particles exhibit insufficient extinction cross sections and larger particles tend to become unstable [21]. Numerous studies have also demonstrated the efficacy of gold nano-flowers (AuNFs) as antibody carriers and detectable markers in LFIA, achieving significantly lower limits of detection (LODs), i.e., 4 to 10 times lower than that of spherical gold nanoparticles (AuNPs) [22,23]. However, these investigations have been constrained by focusing on a single gold nano-flower preparation method, without thoroughly validating the nanoparticle size and requirements or the rationale behind the chosen synthesis method. Gold nano-stars (AuNSs) are also anisotropic particles that possess higher extinction coefficient and higher surface area–volume ratio as compared to spherical nanoparticles, which makes them advantageous in applications like biosensing. Their increased surface area, due to their branched structure, and sharp tips, which act as hotspots, facilitate more effective interactions with biomolecules, boosting their performance in nanoscale processes [24]. Studies have demonstrated the application of gold nano-stars in LFIA for the detection of *Yersinia Pestis* [15] and procalcitonin, a model protein antigen [25].

**Figure 1:**
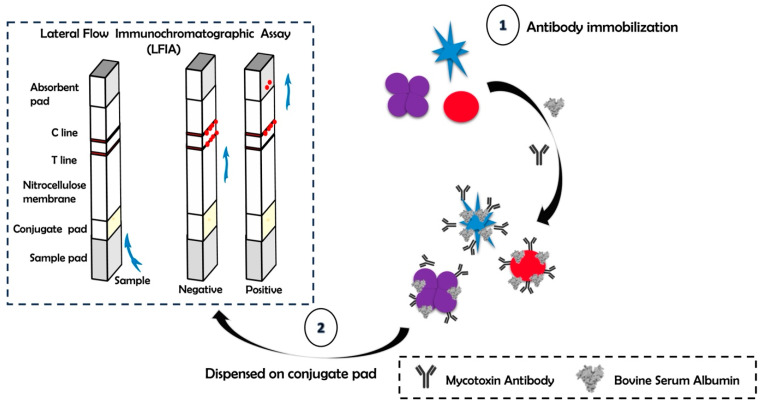
Schematic for development of lateral flow immunochromatographic assay using different gold nano-labels.

Herein, different nano-labels—spherical gold nanoparticles (AuNPs), gold nano-flowers (AuNFs) and gold nano-stars (AuNSs)—have been prepared to assess their sensitivity in the competitive LFIA format for the detection of aflatoxin B1 in food samples, as shown in Figure 1. The developed gold nanoparticle-based LFIA operates on the same detection principle as the traditional competitive LFIA [26]. In the absence of AFB1, the gold nanoparticle–antibody probes are captured by the AFB1-BSA conjugates, resulting in a distinct band at the T line. Conversely, in the presence of AFB1, the gold nanoparticle–antibody probes specifically bind to the target AFB1 in the sample solution, forming a complex. This interaction reduces the capture of gold nanoparticle–antibody conjugates at the T line, leading to a significant decrease in or disappearance of the red band at the T line. The gold nanoparticle-based labels were developed by first immobilizing them with anti-aflatoxin monoclonal antibodies (mAb) by optimizing pH and gold nanoparticle-to-antibody ratio to generate a stable conjugate. LFIA strips were assembled, and the sample pad and conjugate pad were treated with blocking agent and buffer solution to prevent the non-specific binding and effective release of sample from solution. Synthesis of aflatoxin B1–bovine serum albumin (AFB-BSA) hapten was carried out using the oxime active ester (OAE) method [22] and was dropped as test line on the nitrocellulose membrane by using Biodot XYX3060. Aflatoxin B1 standard solutions were prepared at concentrations ranging from 100 µg/mL to 0.01 ng/mL in PBS buffer (0.01M, pH 7.4) with 10% methanol and were run as a sample to assess the sensitivity of different nano-labels. Gold nano-stars, nano-flowers and spherical nanoparticles were assessed based on their sensitivity towards detection of AFB1 in spiked samples. The respective calibration curve was constructed based on the analysis of test line and control line intensities using ImageJ software (Fiji ImageJ 1.51j 2018).

Recent study has also employed different nano-labels for the detection of procalcitonin using lateral flow assay [22] and has shown their efficacy in achieving a LOD OF 0.1 ng/mL in spiked buffer system using nano-popcorns. But this study advances the field by applying gold nano-star-based LFIA to the challenging detection of a small-molecule mycotoxin, achieving higher sensitivity and broader applicability than the previously reported LFIAs. The combination of ultrasensitive detection, practical matrix validation and detailed nanoparticle characterization positions this study as a novel and application-driven contribution to the development of next-generation LFIA platforms for food safety and environmental health.

The proposed approach was demonstrated for the development of competitive LFIA using gold nano-stars for detecting AFB1 in cereal and cereal-based products [23]. Toxic compounds predominantly produced by Aspergillus species contaminate grains under specific environmental conditions [27]. Mycotoxin contamination represents a pressing global challenge, leading to substantial economic losses, with approximately 25% of the food crops worldwide impacted, resulting in an estimated loss of one billion tons of foodstuffs [28]. Due to their toxicological effects, AFB1 in particular is subject to strict regulation by the European Union, necessitating its monitoring as a part of food safety assessment [29]. Hence, the development of analytical devices capable of detecting mycotoxins is critically important to strengthen food quality and safety control strategies throughout the entire production chain [30]. The performance of the gold nano-star LFIA was therefore evaluated in the more complex environment of a wheat matrix spiked with aflatoxin B1 solution.

## 2. Materials and Methods

### 2.1. Materials

Gold (III) chloride trihydrate (HAuCl_4_·3H_2_O), trisodium citrate dihydrate (Na_3_C_6_H_5_O_7_·2H_2_O), sodium hydroxide (NaOH), HEPES buffer (N-(2-hydroxyethyl)piperazine-N′-(2-ethanesulfonic acid)) and hydroquinone were obtained from Sigma-Aldrich, Dublin, (Ireland), along with aflatoxin B1 (AFB1) and carboxymethoxylamine hemi-hydrochloride (CMO). Anti-aflatoxin B1 and anti-mouse IgG monoclonal antibodies were sourced from Abcam, Dublin, (Ireland). Deionized water (DI) used throughout the study was produced using an Elix Reference Water Purification System (Millipore, Dublin, Ireland). All reagents were used as received, without further purification.

### 2.2. Instrumentation

Dynamic light scattering analysis was performed on Zetasizer Nano ZS Analyzer from Malvern Instruments, (Worcestershire, UK), to measure the hydrodynamic particle size, polydispersity index and zeta potential of different formations of GNPs. Spectroscopic analysis of synthesized gold nanoparticles was performed using a Perkin Elmer Lambda 900 UV–visible spectrophotometer procured from PKI scientific Ireland Ltd. Dublin, Ireland and the UV-VIS/NIR spectrum was recorded in the range of 300–800 nm. Transmission electron microscopy (TEM, JEOL2100) procured from Tokyo, Japan, was employed for the morphological evaluation of the synthesized gold nanoparticles. The nanoparticle solution was dispersed in ethanol and DI water, drop-casted on the copper grids, and then air-dried for a few minutes. The images were taken at an accelerating voltage of 120–200 kV.

### 2.3. Synthesis of Spherical Gold Nanoparticles (AuNPs)

The method used to prepare colloidal gold nanoparticles was based on the protocol developed by Borse et al. [9], with minor modifications. A 20 mM stock solution of HAuCl_4_ and 1% trisodium citrate (TSC) was made in deionized water. A final HAuCl_4_ concentration of 0.01% was achieved by mixing 1 mL of 1% HAuCl_4_ with 99 mL of Milli-Q water in a 250 mL conical flask. The mixture was properly mixed and continuously stirred while refluxing at 90 °C. Promptly, 1 mL of 1% TSC was added to the flask while continuously stirring. The initial light-yellow solution turned colorless and bluish grey after around two to five minutes, then turned reddish-purple after an additional five minutes, signifying the formation of AuNPs. After agitating the mixture for ten more minutes, the solution was allowed to cool at room temperature and was stored at 4 °C for further use.

### 2.4. Synthesis of Gold Nano-Flowers (AuNFs)

For the synthesis of gold nano-flowers, the protocol of Zhang et al. [31] was used, with slight modifications. Spherical gold seeds (15 nm) were prepared using 1% sodium citrate and 100 mM HAuCl_4_. Then, 40 µL of HAuCl_4_ was added to 20 mL of DI (deionized) water under stirring and heated to boil. After that, 800 µL of 1 w/v% of sodium citrate was added to the reaction, and the color changed from grey to wine red, indicating the formation of gold nano-seeds [32]. A 30 mM solution of hydroquinone solution was prepared in 10 mL of deionized water. At first, 25 µL of 100 mM HAuCl_4_ was mixed in 9 mL of DI water under vigorous stirring. Subsequently, 200 µL of gold seeds, 22 µL of 1% sodium citrate and 1 mL of 30 mM hydroquinone were added sequentially to the reaction mixture. The mixture was stirred at room temperature for 30 min to obtain a purple color solution, indicating the formation of gold nano-flowers. After agitating the mixture for ten more minutes, the solution was allowed to cool to room temperature and was stored at 4 °C for further use.

### 2.5. Synthesis of Gold Nano-Stars (AuNSs)

Gold nano-stars were prepared according to a one-pot synthesis approach using HEPES buffer, as described by Mulder et al. [33]. In this reaction mixture, 2 mL of 100 mM HEPES solution was mixed with 3 mL of deionized water, followed by the addition of 50 µL of 20 mM HAuCl_4_. The solution was left untouched for 30 min, as the color developed from grey to dark pink and then blue, indicating the formation of gold nano-stars. The solution was then covered and stored at 4 °C for further use.

### 2.6. Preparation of AuNPs/AuNSs/ AuNFs Bioconjugates

To prepare stable gold nanoparticle–antibody conjugates, the pH should be adjusted to the isoelectric point of the protein, and for each antibody, a different pH is required [34]. The AFB1-mAb AuNPs/AuNSs/AuNFs conjugates were created via electrostatic adsorption [35] at the optimum pH value of each of the different gold nanoparticles. Initially, AuNP solutions of varying pH were prepared by adding 0.5, 1, 1.5, 2, 2.5, 4, 5 and 8 μL of 0.2 M solution of K_2_CO_3_ to different vials of 1 mL of gold nanoparticle solutions. The optimum pH value for the aflatoxin antibody was determined from the study [36]. Using the checkerboard titration method [37], AFB1-mAb solutions, with a volume of 2, 4, 8, 10 and 20 μL, were introduced to the AuNPs/AuNSs/AuNFs at pH 7.5 in the different wells of a 96-well plate to determine the optimum amount of antibodies required to completely cover the surface of gold nanoparticles, so they maintain their stability when exposed to an alkaline environment resisting aggregation. The solution was then mixed for a duration of 30 min. After that, 100 μL of 10% NaCl was added to the conjugate solutions, mixed evenly and kept for 15 min. Spectrophotometric analysis in the range 300–800 nm was performed to verify the ideal concentration of antibody along with visual inspection for color change and aggregation. The minimum antibody required to cover the particle surface sufficiently to prevent aggregation in the presence of NaCl was chosen. Following the addition of an optimized quantity of antibodies, the remaining active surface was blocked by incubating with 100 μL of 10% bovine serum albumin (BSA) for 30 min. Subsequently, the reaction mixture was centrifuged at 8000 rpm for 15 min to remove the supernatant. The antibody binding efficiency was calculated using the Bradford assay [38], calculating the amount of protein in the supernatant using the calibration curves in Supplementary Figures S1 and S2. The resulting gold nanoparticle–antibody conjugates were resuspended in 1 mL of phosphate-buffered saline (PBS, 0.01M, pH 8.5) containing 0.1% BSA and 0.1% sucrose, to preserve the secondary structure of proteins, and stored at 4 °C for future use.

### 2.7. Synthesis of Aflatoxin–BSA Hapten

The synthesis of the AFB-BSA hapten was conducted using the oxime active ester (OAE) method [39]. Initially, 5 mg of AFB1 was dissolved in 3 mL of pyridine, along with 12 mg of carboxymethoxylamine hemi-hydrochloride. The reaction mixture was stirred and sealed for 24 h in the dark. Subsequently, the mixture was freeze-dried to yield AFB1 oxime as a white powder. For the hapten activation step, 3 mg of AFB1-oxime was combined with 2 mg of EDC (1-ethyl-3-(3-dimethylaminopropyl) carbodiimide in 1 mL of dimethylformamide (DMF) and incubated in the dark for 3 h. A solution of BSA was prepared in advance at a concentration of 10 mg/mL. The activated hapten solution was then added dropwise to the BSA solution and maintained at room temperature for 2 h. Finally, the resulting solution was dialyzed against 0.01 M PBS buffer for 3 days, and any precipitate was removed by centrifugation and stored at 4 °C for further use.

### 2.8. Assembling Lateral Flow Assay Immunochromatographic Assay (LFIA) Strips

Lateral flow immunoassay (LFIA) strips were assembled using a sample pad, conjugate pad, nitrocellulose membrane and absorbent pad. Prior to assembly, the sample and conjugate pads were subjected to pre-treatment to equilibrate the membranes, prevent pore fouling and ensure consistent and efficient sample and conjugate migration across the strip [40]. All fabrication and pre-treatment procedures were conducted under sterile conditions in a laminar flow chamber. The conjugate pad was immersed in a 50 mM phosphate-buffered saline (PBS) solution containing 0.1% bovine serum albumin (BSA), 5% sucrose and 0.05% Tween-20, followed by drying at 60 °C for 1 h. Similarly, the sample and absorbent pads were treated with 50 mM PBS supplemented with 0.1% BSA, 5% sucrose, 0.01 g sodium azide and 0.05% Tween-20. The test line was prepared by dispensing 0.3 mg/mL AFB-BSA hapten solution, and the control line by applying 0.5 mg/mL anti-mouse IgG onto the nitrocellulose membrane. Subsequently, 10 µL of AuNP-, AuNS- or AuNF-conjugated monoclonal antibody solution was dispensed onto the pre-treated conjugate pad and dried at 37 °C for 2 h. Assembled LFIA strips were stored at room temperature in sealed plastic pouches until further use.

### 2.9. Analytical Performance of LFIA

All the aflatoxin B1 standard solutions were made at concentrations from 100 µg/mL to 0.01 ng/mL in PBS buffer (0.01M, pH 7.4) with 10% methanol [41]. A test strip was positioned on a horizontal surface, and 100 µL of different concentrations of aflatoxin B1 standard solution was applied to the sample pad to conduct the lateral flow immunoassay. Each standard solution was analyzed in triplicate. The intensities of the colored test line and control line on the strips were measured after 10 min. The image of the strips was taken under white light, and the line intensities were digitized using ImageJ software. The calibration curve was plotted using the color response T, relative to the unexposed response T_0_, plotted as a function of the exposure concentration (C) along with the corresponding regression equation for a semi-quantitative assay developed.

### 2.10. Spiked Sample Analysis in Wheat Matrix

Wheat samples free from aflatoxin B1 were collected from a nearby grain procurement market from Dublin and were preprocessed for the analysis as per protocol [42]. Briefly, 5g of wheat was spiked with varying volumes of AFB1(0, 0.5, 1, 2.5, 5, 10 µg/kg). The sample was then ground into fine powder and mixed with 20 mL of methanol–water mixture (80:20, v/v). The extract was then filtered to remove the large insoluble particles, followed by centrifugation at 3000 rpm for 5 min. The supernatant was then collected and diluted five times with phosphate-buffered saline with Tween-20 (PBST) (0.01M, pH 7.4, 0.1% Tween-20) for detection. Then, 100 µL of solution was added to the sample pad for analysis on LFIA with AuNSs as nano-labels, and a calibration curve was plotted for the concentration of AFB1 against T/T_0_ value.

## 3. Results and Discussion

### 3.1. Characterization of Hierarchical Nano-Labels

**Figure 2:**
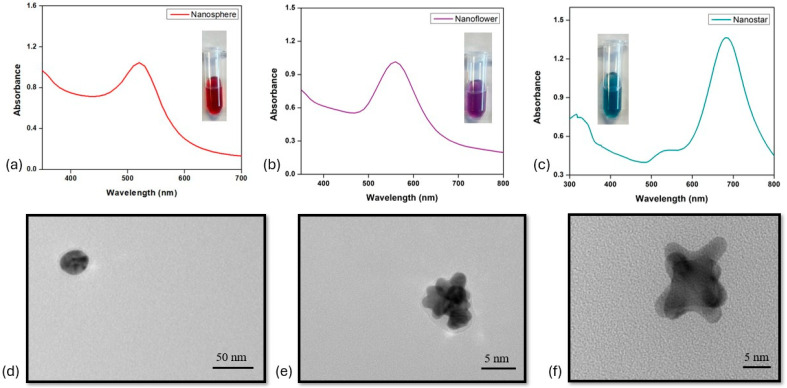
Characterization of gold nanoparticles. (**a**) UV-VIS/NIR spectra for spherical gold nanoparticles (AuNPs). (**b**) UV-VIS/NIR spectra for gold nano-flowers (AuNFs). (**c**) UV-VIS/NIR spectra of gold nano-stars (AuNSs). (**d**) TEM micrograph of spherical gold nanoparticle. (**e**) TEM micrograph of gold nano-flower. (**f**) TEM micrograph of gold nano-star.

The physicochemical properties, notably the color, of the gold nanoparticles depend upon their shape and size [43]. Figure 2 shows the UV-VIS/NIR spectra and transmission electron microscopy (TEM) images for the synthesized gold nanoparticles. The characteristics peak and λ_max_ of bare spherical gold nanoparticles is at 526 nm, for gold nano-flowers at 559 nm and for branched nano-stars at 681 nm. The morphological data from TEM clearly depict the formation of spherical, flower- and star-shaped gold nanoparticles in Figure 2d–f. The hydrodynamic size of the synthesized nanoparticles was determined using dynamic light scattering (DLS), as shown in Figure 1 and detailed in Table 1. With diameters of spherical nanoparticles of 33.2 ± 1.5 nm, nano-stars of 30.7 ± 1.25 nm and nano-flowers of 40.7 ± 1.8 nm, all the synthesized particles exhibit good colloidal stability and homogeneity in terms of size and composition. To confirm the stability and reproducibility of synthesized gold nano-labels, the measurements were taken in triplicate (n = 3) from each batch, and the data are shown in Supplementary Figure S1.

**Figure 3:**
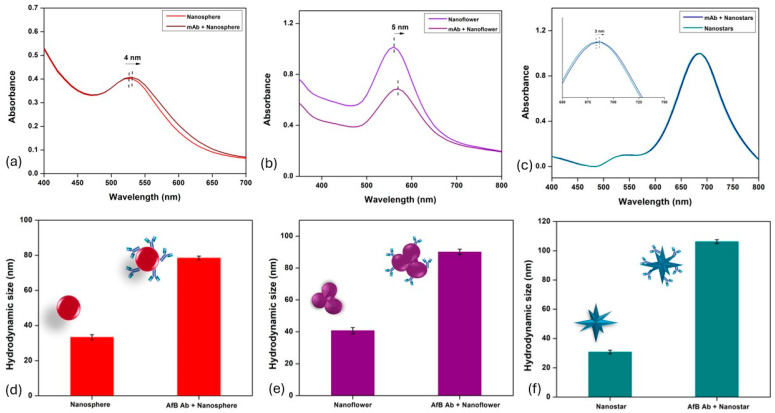
Change in UV-VIS/NIR spectroscopy and hydrodynamic sizes (nm) of gold nanoparticles after the attachment of anti-aflatoxin B1 antibodies. (**a**) UV-VIS/NIR spectra of AuNPs and AuNPs-mAb. (**b**) UV-VIS/NIR spectra of AuNFs and AuNFs-mAb. (**c**) UV-VIS/NIR spectra of AuNSs and AuNSs-mAb. (**d**) Hydrodynamic size of AuNPs and AuNPs-mAb. (**e**) Hydrodynamic size of AuNFs and AuNFs-mAb. (**f**) Hydrodynamic size of AuNSs and AuNSs-mAb.

After the immobilization of aflatoxin antibody (AFB-mAb) onto these synthesized gold nanoparticles, the change in size and zeta potential of the conjugates was determined using DLS to be 78.4 ± 1.8 nm for AuNPs-mAb, 90.1 ± 1.7 nm for AuNFs-mAb and 106.2 ± 1.3 nm for AuNSs-mAb, as shown in Figure 3d–f. Changes in the UV-VIS/NIR spectra were also observed as a red shift, which occurs due to the change in refractive index of the particle’s surroundings [40], and this was caused by the attachment of antibodies on the biochemical corona layer on the particle surface. For spherical nanoparticles (Figure 3a), a shift in the maximum wavelength (λ_max_) of 4 nm is observed, i.e., from 526 nm to 530 nm. Similarly, nano-flowers in Figure 3b exhibit a 5 nm shift in λ_max_, from 559 nm to 564 nm, and nano-stars in Figure 3c show a 3 nm shift in λ_max_, from 681 nm to 684 nm. The amount of antibodies on the respective gold nano-labels was quantified using the protein Bradford assay along with the immobilization efficiency of AFB1 antibodies for each gold nanoparticle. Supplementary Figure S2 shows the standard curve for BSA protein, which was used to calculate the amount of antibody immobilized onto the gold nanoparticles. The linear curve plotted shows the equation y = 0.0568x + 0.130 with R^2^ = 0.992. Supplementary Figure S3 shows the amount of antibody immobilized for each of the different gold nanoparticles and their immobilization efficiency was calculated based on the difference in total volume of antibodies added to the solution and volume in supernatant. The maximum immobilization efficiency of 86% was observed for gold nano-stars (AuNSs) due to their high surface-to-volume ratio, compared to nano-flowers (AuNFs) with 81% and spherical nanoparticles (AuNPs) with 83% efficiency. The concentrations of the anti-aflatoxin antibody determined as optimal to prepare the stable conjugates were different for each of the particle types; 8 µg/mL for AuNPs, 6 µg/mL for AuNFs and 10 µg/mL for AuNSs. Compared to gold nano-stars, gold nano-flowers exhibited greater stability and did not show any visible color change at an antibody concentration of 6 µg/mL. Even at higher concentrations (8–10 µg/mL), the conjugates remained stable; however, increasing antibody concentration also raises the overall cost of the assay. Therefore, the minimum antibody concentration necessary to achieve stable conjugation was selected to balance performance with cost efficiency. This indicates that the multibranched gold nano-stars require a higher antibody concentration to form a stable conjugate, followed by spherical nanoparticles and nano-flowers. Although the surface area was not explicitly measured but having comparable hydrodynamic diameters, gold nano-stars have higher surface area due to their unique branched morphology. AuNSs have multiple branches and sharp tips. These structural features greatly increase the surface area and create regions of high surface curvature, which enhance their interaction with surfaces and biomolecules [15], thereby increasing their capacity to immobilize more antibodies. In contrast, spherical nanoparticles have smooth, isotropic surfaces, offering the lowest surface-to-volume ratio among the three morphologies [44]. Gold nano-flowers possess more irregular and textured surfaces than spheres, but their protrusions are generally broader and less defined than nano-stars, resulting in a smaller increase in surface area as compared to nano-stars [45].

### 3.2. Optimization of LFIA Procedure

For the comparative analysis and detection of aflatoxin B1 in samples, gold nanoparticles of each type (AuNPs, AuNFs and AuNSs) were utilized as labels in LFIA. The assay system optimization included selecting specific antibodies, determining the concentration of gold nanoparticle–antibody conjugates and establishing the appropriate concentrations of immunoreagents for the control and test lines. To determine the optimal dilution of gold nanoparticle–antibody conjugates for lateral flow assay, the optical density (OD) of the solutions at a specific wavelength was measured to ensure sufficient color intensity developed at the test line and control line. The test line hapten was characterized by UV-VIS spectroscopy, and the data are shown in Supplementary Figure S4. For each nanoparticle type, this was determined to be at an optical density (OD) of 2. The gold nanoparticle–antibody conjugates were impregnated in the conjugate pad. The strips were also optimized based on coating the nitrocellulose membrane with BSA solution. Supplementary Figure S5a shows the difference in treated vs non-treated nitrocellulose membrane with buffer and surfactant solution for effective running of sample solution on the membrane. The color developed on the background of membrane on untreated strips can hinder the scanning and digitizing process. However, on coating them with 0.1% BSA in borate buffer, the nanoparticles did not deposit on the membrane, leaving a clear background for the measurement, as shown in Supplementary Figure S5b. The optimal concentrations of the immobilized IgG and toxin–protein hapten on control line and test line were, respectively, determined to be 0.5 mg mL^−1^ and 0.3 mg mL^−1^.

### 3.3. Comparison of Analytical Performance and Sensitivity for Different Nano-Labels and Spiked Sample Analysis

**Figure 4:**
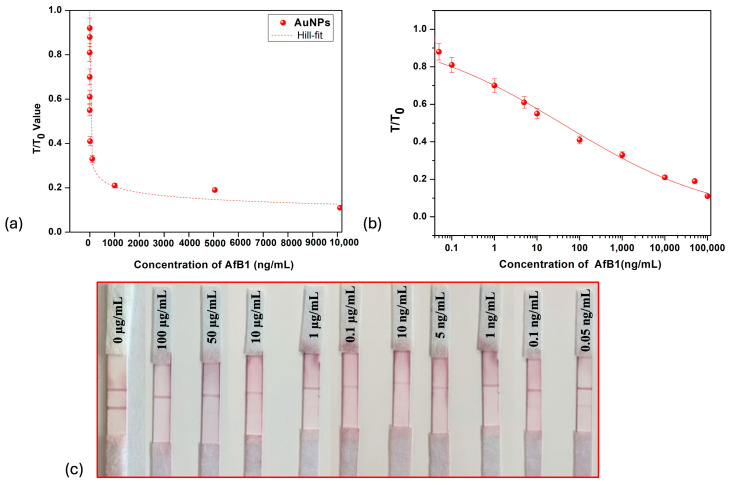
Gold nanoparticles (AuNPs) as nano-labels. (**a**) T/T_0_ intensity value vs concentration of aflatoxin B1 for gold nanoparticles. (**b**) Linear–log curve for AuNPs. (**c**) LFIA strips for different concentrations of aflatoxin B1.

**Figure 5:**
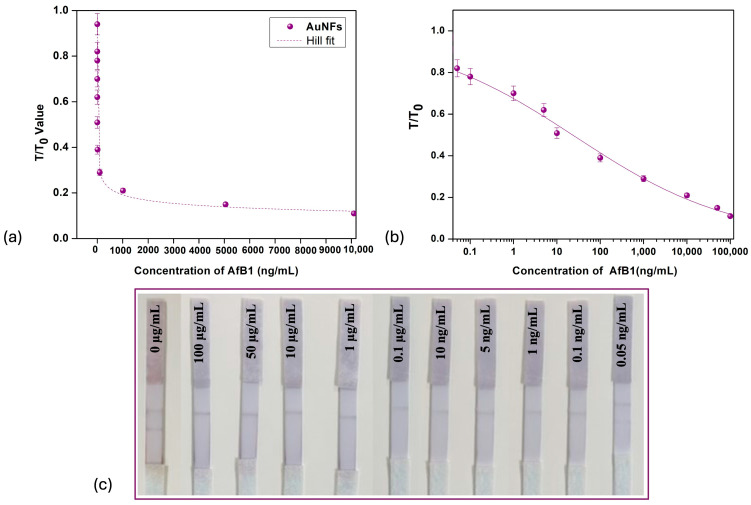
Gold nano-flowers (AuNFs) as nano-labels. (**a**) T/T_0_ intensity value vs concentration of aflatoxin B1 for gold nano-flower. (**b**) Linear–log curve for AuNFs. (**c**) LFIA strips for different concentrations of aflatoxin B1.

**Figure 6:**
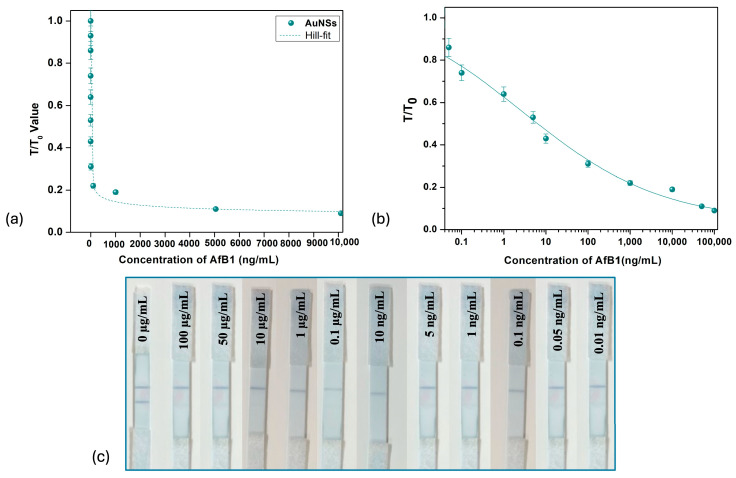
Gold nano-stars (AuNSs) as nano-labels. (**a**) T/T_0_ intensity value vs concentration of aflatoxin B1 for gold nano-stars. (**b**) Linear–log curve for AuNSs. (**c**) LFIA strips for different concentrations of aflatoxin B1.

The analytical conditions were optimized to enable an accurate comparative evaluation of the three different gold nanoparticle preparations in LFIA. Figure 4 shows the LFIA strips using spherical gold nanoparticle (AuNPs) when they were analyzed for their sensitivity in detecting aflatoxin B1. The color developed after treating the strips with the positive sample was highest and most vibrant when spherical AuNPs were employed as labeling agents. The visual limit of detection achieved was 0.1 ng/mL. Figure 5 shows the sensitivity of gold nano-flower LFIAs assessed for AFB1 detection, achieving a visual LOD of 0.1 ng/mL. It was found that the highest sensitivity was achieved with gold nano-star AuNSs (Figure 6), with the lowest detection limit (LOD) of 0.01 ng/mL, consistent with the previous reports [46]. On the other hand, in the case of AuNPs and AuNFs, the limit of detection was 0.1 ng/mL as visualized by the naked eye. The use of multibranched AuNSs improved the sensitivity approximately five-fold compared to AuNPs and AuNFs. The comparison between gold nanoparticles, nano-stars and nano-flowers indicates that the shape rather than particle size affected the sensitivity of the analysis [19,47].

The color developed on the test line was brightest when spherical gold nanoparticles (red) were employed, followed by gold nano-stars (blue), and was lowest for nano-flowers (purple) (AuNPs > AuNSs > AuNFs). Although gold nano-stars possess an extinction coefficient higher than that of gold nanospheres, the LSPR band of gold nano-stars are in the near-infrared (NIR) region, where visual inspection is ineffective. For AuNPs, the strips were exposed to analyte concentrations diluted until the limit of detection of 0.05 ng/mL was reached, at which one can clearly still see the slight color development on the test line. Figure 4a shows the graph of the ratio of line intensities (T/T_0_) versus concentration of aflatoxin for the case of spherical gold nanoparticles (AuNPs). In competitive lateral flow assay, the graph of T/T_0_ vs analyte concentration depicts an inverse relationship; as the concentration of analyte increases, more analyte molecules occupy the binding site, reducing the ability of gold nanoparticle labeled molecules to bind, thus reducing the relative intensity of the test line readout (T). Similarly, at low analyte concentration, fewer analyte molecules compete with labeled molecules for binding, such that the value of T asymptotically approaches the value of T_0_ [48].

This binding should follow a Langmuir isotherm-like behavior [49], although other factors should be considered, such as the amount of gold nanoparticle–antibody conjugate binding to the control line, where IgG is present. The Hill equation models cooperative binding of ligands to receptors or adsorption sites, and it extends the Langmuir isotherm by introducing the Hill coefficient ‘*n*’, which quantifies cooperativity. *C* is the concentration of the absorbate, *K* is the dissociation constant (analogous to Langmuir’s constant) and *n* is Hill’s coefficient, so the fraction of the occupied sites is given by fractional binding (θ),

θ(*C*) = *C^n^*/*K^n^* +*C^n^*

this equation describes how adsorption saturates with increasing concentration, adjusted by cooperativity. Now mapping θ to T/T_0_ response, x (0) is the baseline response when *C* = 0, x (sat) is the saturated response at high *C*, and we have used θ to interpolate between x (0) and x (sat) using

f(*C*) = x (0) + (x (sat) − x (0)) × θ (*C*)

substituting for θ, we get,

f(*C*) = x (0) + (x (sat) − x (0)) × *C^n^* (*K^n^* + *C^n^*)

The T/T_0_ plots in Figure 4a can be well fitted with a Hill-like equation of the form where x (0) is the unexposed value of T/T_0_; x (sat) is the highest concentration, saturated value; *n* is the Hill slope; and *K* is the equivalent of the Langmuir constant, a measure of how strongly an adsorbate molecule interacts with a specific site on the surface. In Figure 4b, for the better visualization of the lower-concentration region, the graph shows approximate linearity within the range from 0.05 ng/mL to 100 ng/mL.

Similar behaviors are observed in Figure 5a and Figure 6a for the cases of AuNFs and AuNSs, respectively, and the parameters for the Hill-fit for all the nano-labels employed are summarized in Table 2. Compared to spherical gold nanoparticles and gold nano-flowers, nano-stars achieved a lower K value, indicating that a lower concentration of AFB1 is required to cause a significant response (i.e., half maximal signal change), in turn implying higher binding affinity and enhanced sensitivity. This result is consistent with the observation that the lowest LOD was achieved using gold nano-stars (AuNSs), compared to spherical and flower-shaped gold nanoparticles. The larger surface area of the gold nano-stars leads to adsorption of more antibodies, enhancing the sensitivity and signal intensity [50]. Compared to spherical nanoparticles, gold nano-stars are generally considered more stable as the irregular geometry reduces Van Der Waals interactions, making them less prone to clumping in a biological environment, which maintains their consistent performance in assays, reducing variability and thereby improving reliability [51].

Gold nano-stars (AuNSs) exhibit superior signal enhancement in LFIA due to their unique branched morphology. The sharp tips create localized electric fields that reduce electrostatic repulsion between the negatively charged gold surface and antibody molecule, effectively enhancing charge shielding and conjugation efficiency [51]. The anisotropic surface promotes favorable antibody orientation, improving antigen accessibility and strengthening antigen binding via avidity effects, resulting in amplified test signals [52,53,54].

**Figure 7:**
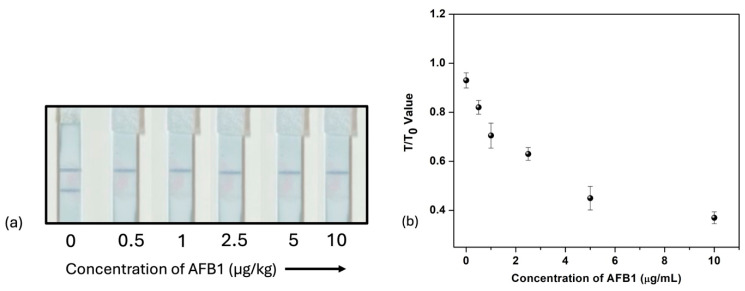
(**a**) Responses of gold nano-star (AuNS)-labeled LFIA in detection of AFB1 spiked samples in wheat matrix (n = 3). (**b**) Calibration curve for concentration vs. T/T_0_ response.

To further verify the feasibility of establishing LFIA using gold nano-stars as labels, aflatoxin B1 was detected in spiked samples of a wheat matrix. The spiked samples of different concentrations were dropped onto respective sample pads for visual naked-eye measurement. As shown in Figure 7a, the test line intensity decreased as the concentration of AFB1 was increased in the solution. The graph in Figure 7b also shows that the T/T_0_ ratio on the y-axis and the concentration of AFB1 on the x-axis decrease as the concentration of aflatoxin B1 is increased. The analysis is semi-quantitative, and the result is observed by the presence or absence of a colored line on the test zone of the strips. The time of analysis is less than 10 min and can be performed outside the laboratory, meeting the requirements of point-of-care testing. The developed lateral flow immunoassay demonstrates a limit of detection of 0.05 ng/mL (equivalent to 0.05 µg/L), which is well below the maximum residue limits set by the European Union for aflatoxin B1 in food products [29]. This indicates that the assay is sufficiently sensitive for regulatory compliance and suitable for effective screening of contaminated samples.

## 4. Conclusions

In this study, different types of gold nano-labels were considered and compared to assess the sensitivity of the lateral flow immunoassay, incorporating them into the detection of aflatoxin B1. It was shown that shape and size affect the sensitivity of LFIA. Spherical gold nanoparticles are widely employed as labels in the detection of analytes, but they lack sensitivity. Gold nano-flowers and nano-stars exhibit advantages in the field of sensing and diagnostics due to their enhanced surface area, localized surface plasmon resonance, improved stability and selective binding. Among all the selected nano-labels, gold nano-stars exhibit the highest sensitivity towards aflatoxin B1, with detection limits as low as 0.01 ng/mL. Due to their unique geometry, nano-stars achieve the highest anti-aflatoxin antibody binding efficiency of 86%, which allows for increased sensitivity. A five-fold improvement in the sensitivity with an aflatoxin B1 detection limit of 0.05 ng/mL was achieved using gold nano-stars, compared to the other two nano-labels. LFIA developed using gold nano-stars as a labels was feasible and was able to detect AFB1 even at minute concentrations in a wheat matrix. The permissible limit for aflatoxin B1 under EU regulations in wheat samples for unprocessed cereals is 5 µg/kg and for processed cereal-based products is 2 µg/kg. The developed immunoassay achieves a limit of detection well below the maximum residue limits. The integration of diverse gold nanostructures into lateral flow immunoassays has the potential to significantly enhance the sensitivity for the detection of analytes in food and feed products.

## Figures and Tables

**Table 1:** UV-VIS/NIR peak position (λ_max_), size (nm) and zeta potential of gold nanoparticles upon addition of anti-aflatoxin B1 antibodies.

Type of Gold Nanoparticle	Hydrodynamic Size (nm)	Hydrodynamic Size (nm) After AFB1-mAb Immobilization	Change in UV-VIS/NIR Peak Wavelength (λ_max_)	Change in Zeta Potential (mV)
AuNPs	33.2 ± 1.5 nm	78.4 ± 1.8 nm	526 to 530 nm	−33.7 to −20.7 mV
AuNFs	40.7 ± 1.8 nm	90.1 ± 1.7 nm	559 to 564 nm	−30.3 to −22.4 mV
AuNSs	30.7 ± 1.2 nm	106.2 ± 1.3 nm	681 to 684 nm	−35.2 to −19.6 mV

**Table 2:** Hill-fit parameters and analytical performance of developed LFIA comparison using different nano-labels in aflatoxin B1 detection.

Hill-Fit Parameters	AuNPs	AuNFs	AuNSs
Start (minimum signal)	0.993	0.982	1.07
End (maximum signal)	0.01	0.09	0.03
k (half maximum concentration)	43.97 ng/mL	26.90 ng/mL	2.84 ng/mL
Hill coefficient (n)	0.235	0.239	0.264
Dynamic range	0.1 ng/mL–100 µg/mL	0.1 ng/mL–100 µg/mL	0.01 ng/mL–100 µg/mL
R^2^	0.968	0.987	0.973
LOD	0.1 ng/mL	0.1 ng/mL	0.01 ng/mL

## Data Availability

Data are contained within the article.

## Supplementary Materials

The following supporting information can be downloaded at: https://www.mdpi.com/article/10.3390/bios15080495/s1, Figure S1: Nano label synthesis batch 1 to batch 3 variability in hydrodynamic size (nm), Figure S2: Standard curve for BSA protein for the quantification of immobilization efficiency of nano labels, Figure S3: Amount of anti-aflatoxin antibody immobilized and their immobilization efficiency calculated using Bradford assay, Figure S4: UV-Vis spectroscopy of the synthesized AFB-BSA hapten as immunoreagent for test line, Figure S5: LFIA images treated vs untreated nitrocellulose membrane. (a) untreated with BSA leading to deposition of the nano labels on the membrane; (b) membrane treated with 1% BSA in borate buffer, Table S1: Mean size and CV(%) of synthesized nano labels across 3 batches.

## References

[1] Kakkar S., Gupta P., Yadav S.P.S., Raj D., Singh G., Chauhan S., Mishra M.K., Martín-Ortega E., Chiussi S., Kant K. (2024). Lateral flow assays: Progress and evolution of recent trends in point-of-care applications. Mater. Today Bio.

[2] Bahadır E.B., Sezgintürk M.K. (2016). Lateral flow assays: Principles, designs and labels. TrAC Trends Anal. Chem..

[3] Nguyen V.-T., Song S., Park S., Joo C. (2020). Recent advances in high-sensitivity detection methods for paper-based lateral-flow assay. Biosens. Bioelectron..

[4] Pedreira-Rincón J., Rivas L., Comenge J., Skouridou V., Camprubí-Ferrer D., Muñoz J., O’SUllivan C.K., Chamorro-Garcia A., Parolo C. (2025). A comprehensive review of competitive lateral flow assays over the past decade. Lab A Chip.

[5] Lin Y.-Y., Wang J., Liu G., Wu H., Wai C., Lin Y. (2008). A nanoparticle label/immunochromatographic electrochemical biosensor for rapid and sensitive detection of prostate-specific antigen. Biosens. Bioelectron..

[6] Chen C., Wu J. (2012). A Fast and Sensitive Quantitative Lateral Flow Immunoassay for Cry1Ab Based on a Novel Signal Amplification Conjugate. Sensors.

[7] Goryacheva I.Y., Lenain P., De Saeger S. (2013). Nanosized labels for rapid immunotests. TrAC Trends Anal. Chem..

[8] Beloglazova N.V., Goryacheva I.Y., Niessner R., Knopp D. (2011). A comparison of horseradish peroxidase, gold nanoparticles and qantum dots as labels in non-instrumental gel-based immunoassay. Microchim. Acta.

[9] Borse V., Konwar A.N. (2020). Synthesis and characterization of gold nanoparticles as a sensing tool for the lateral flow immunoassay development. Sensors Int..

[10] Byzova N.A., Zherdev A.V., Khlebtsov B.N., Burov A.M., Khlebtsov N.G., Dzantiev B.B. (2020). Advantages of Highly Spherical Gold Nanoparticles as Labels for Lateral Flow Immunoassay. Sensors.

[11] Huang X., El-Sayed M.A. (2010). Gold nanoparticles: Optical properties and implementations in cancer diagnosis and photothermal therapy. J. Adv. Res..

[12] Omidfar K., Kia S., Kashanian S., Paknejad M., Besharatie A., Kashanian S., Larijani B. (2009). Colloidal Nanogold-Based Immunochromatographic Strip Test for the Detection of Digoxin Toxicity. Appl. Biochem. Biotechnol..

[13] Yu Q., Zhang J., Qiu W., Li K., Qian L., Zhang X., Liu G. (2021). Gold nanorods-based lateral flow biosensors for sensitive detection of nucleic acids. Microchim. Acta.

[14] Liang R., Wang F., Li S., Niu Y., Sun Y., Hong S., Fan A. (2024). A sensitive gold nanoflower-based lateral flow assay coupled with gold staining technique for the detection of SARS-CoV-2 antigen. Microchim. Acta.

[15] Atta S., Canning A.J., Odion R., Wang H.-N., Hau D., Devadhasan J.P., Summers A.J., Gates-Hollingsworth M.A., Pflughoeft K.J., Gu J. (2023). Sharp Branched Gold Nanostar-Based Lateral-Flow Immunoassay for Detection of *Yersinia pestis*. ACS Appl. Nano Mater..

[16] Munyayi T.A., Vorster B.C., Mulder D.W. (2022). The Effect of Capping Agents on Gold Nanostar Stability, Functionalization, and Colorimetric Biosensing Capability. Nanomaterials.

[17] Delgado-Corrales B.J., Chopra V., Chauhan G. (2024). Gold nanostars and nanourchins for enhanced photothermal therapy, bioimaging, and theranostics. J. Mater. Chem. B.

[18] Lai W., Xiong Z., Huang Y., Su F., Zhang G., Huang Z., Peng J., Liu D. (2019). Gold nanoflowers labelled lateral flow assay integrated with smartphone for highly sensitive detection of clenbuterol in swine urine. Food Agric. Immunol..

[19] Petrakova A.V., Urusov A.E., Zherdev A.V., Dzantiev B.B. (2019). Gold nanoparticles of different shape for bicolor lateral flow test. Anal. Biochem..

[20] Khlebtsov B.N., Tumskiy R.S., Burov A.M., Pylaev T.E., Khlebtsov N.G. (2019). Quantifying the Numbers of Gold Nanoparticles in the Test Zone of Lateral Flow Immunoassay Strips. ACS Appl. Nano Mater..

[21] Kim D.S., Kim Y.T., Hong S.B., Kim J., Heo N.S., Lee M.-K., Lee S.J., Kim B.I., Kim I.S., Huh Y.S. (2016). Development of Lateral Flow Assay Based on Size-Controlled Gold Nanoparticles for Detection of Hepatitis B Surface Antigen. Sensors.

[22] Serebrennikova K.V., Samsonova J.V., Osipov A.P., Senapati D., Kuznetsov D.V. (2017). Gold Nanoflowers and Gold Nanospheres as Labels in Lateral Flow Immunoassay of Procalcitonin. Nano Hybrids Compos..

[23] Wang R., Wang J., Liu H., Gao Y., Zhao Q., Ling S., Wang S. (2021). Sensitive immunoassays based on specific monoclonal IgG for determination of bovine lactoferrin in cow milk samples. Food Chem..

[24] D P.N., Sinha R.K. (2024). Shape-controlled Synthesis and Bulk Refractive Index Sensitivity Studies of Gold Nanoparticles for LSPR-based Sensing. Plasmonics.

[25] Atta S., Zhao Y., Sanchez S., Seedial D., Devadhasan J.P., Summers A.J., Gates-Hollingsworth M.A., Pflughoeft K.J., Gu J., Montgomery D.C. (2024). Plasmonic-Enhanced Colorimetric Lateral Flow Immunoassays Using Bimetallic Silver-Coated Gold Nanostars. ACS Appl. Mater. Interfaces.

[26] Prakashan D., Kolhe P., Gandhi S. (2023). Design and fabrication of a competitive lateral flow assay using gold nanoparticle as capture probe for the rapid and on-site detection of penicillin antibiotic in food samples. Food Chem..

[27] Açar Y., Akbulut G. (2024). Evaluation of Aflatoxins Occurrence and Exposure in Cereal-Based Baby Foods: An Update Review. Curr. Nutr. Rep..

[28] Krska R., Schubert-Ullrich P., Molinelli A., Sulyok M., MacDonald S., Crews C. (2008). Mycotoxin analysis: An update. Food Addit. Contam. Part A.

[29] Neme K., Mohammed A. (2017). Mycotoxin occurrence in grains and the role of postharvest management as a mitigation strategies. A review. Food Control.

[30] Sadeghi P., Sohrabi H., Majidi M.R., Eftekhari A., Zargari F., de la Guardia M., Mokhtarzadeh A.A. (2024). Mycotoxins detection in food samples through lateral flow assays (LFAs)—An update for status and prospect. TrAC Trends Anal. Chem..

[31] Anfossi L., Giovannoli C., Baggiani C. (2016). Mycotoxin detection. Curr. Opin. Biotechnol..

[32] Zhang W., Duan H., Chen R., Ma T., Zeng L., Leng Y., Xiong Y. (2019). Effect of different-sized gold nanoflowers on the detection performance of immunochromatographic assay for human chorionic gonadotropin detection. Talanta.

[33] Li J., Wu J., Zhang X., Liu Y., Zhou D., Sun H., Zhang H., Yang B. (2011). Controllable Synthesis of Stable Urchin-like Gold Nanoparticles Using Hydroquinone to Tune the Reactivity of Gold Chloride. J. Phys. Chem. C.

[34] Mulder D.W., Phiri M.M., Jordaan A., Vorster B.C. (2019). Modified HEPES one-pot synthetic strategy for gold nanostars. R. Soc. Open Sci..

[35] Ackerson C.J., Jadzinsky P.D., Jensen G.J., Kornberg R.D. (2006). Rigid, Specific, and Discrete Gold Nanoparticle/Antibody Conjugates. J. Am. Chem. Soc..

[36] Busch R.T., Karim F., Weis J., Sun Y., Zhao C., Vasquez E.S. (2019). Optimization and Structural Stability of Gold Nanoparticle–Antibody Bioconjugates. ACS Omega.

[37] Sharma V., Javed B., Byrne H.J., Tian F. (2024). Mycotoxin Detection through Colorimetric Immunoprobing with Gold Nanoparticle Antibody Conjugates. Biosensors.

[38] Zhou H., He C., Li Z., Huo J., Xue Y., Xu X., Qi M., Chen L., Hammock B.D., Zhang J. (2022). Development of a Rapid Gold Nanoparticle Immunochromatographic Strip Based on the Nanobody for Detecting 2,4-DichloRophenoxyacetic Acid. Biosensors.

[39] Tabassum H., Maity A., Singh K., Bagchi D., Nath P., Kumar N., Choudhury S., Vishwakarma S., Chakraborty A. (2025). Elucidating Antibody Conjugation and Orientation Dynamics on Phenylalanine-Functionalized Gold Nanoparticles: The Role of Lipid Coating and Other Physiological Conditions. Langmuir.

[40] Han S., Yang Y., Chen T., Yang B., Ding M., Wen H., Xiao J., Cheng G., Tao Y., Hao H. (2024). Quantitative Determination of Aflatoxin B_1_ in Maize and Feed by ELISA and Time-Resolved Fluorescent Immunoassay Based on Monoclonal Antibodies. Foods.

[41] Sukumaran A., Thomas T., Thomas R., Thomas R.E., Paul J.K., Vasudevan D.M. (2020). Development and Troubleshooting in Lateral Flow Immunochromatography Assays. Indian J. Clin. Biochem..

[42] Wang D., Zhu J., Zhang Z., Zhang Q., Zhang W., Yu L., Jiang J., Chen X., Wang X., Li P. (2019). Simultaneous Lateral Flow Immunoassay for Multi-Class Chemical Contaminants in Maize and Peanut with One-Stop Sample Preparation. Toxins.

[43] Zhang Y., Chen G., Chen X., Wei X., Shen X.-A., Jiang H., Li X., Xiong Y., Huang X. (2024). Aggregation-induced emission nanoparticles facilitating multicolor lateral flow immunoassay for rapid and simultaneous detection of aflatoxin B1 and zearalenone. Food Chem..

[44] Sharma V., Javed B., Estrada G., Byrne H.J., Tian F. (2023). In situ tuning and investigating the growth process of size controllable gold nanoparticles and statistical size prediction analysis. Colloids Surfaces A Physicochem. Eng. Asp..

[45] Siegel A.L., Baker G.A. (2021). Bespoke nanostars: Synthetic strategies, tactics, and uses of tailored branched gold nanoparticles. Nanoscale Adv..

[46] Daniel M., Astruc D. (2004). Gold Nanoparticles: Assembly, Supramolecular Chemistry, Quantum-Size-Related Properties, and Applications toward Biology, Catalysis, and Nanotechnology. Chem. Rev..

[47] Khakbiz M., Shakibania S., Ghazanfari L., Zhao S., Tavakoli M., Chen Z. (2023). Engineered nanoflowers, nanotrees, nanostars, nanodendrites, and nanoleaves for biomedical applications. Nanotechnol. Rev..

[48] Wu Y., Yang Q., Chen J., Bi L., Zhang Z., Zhou N., Ostovan A., Arabi M., Chen L., Choo J. (2024). Surface-Enhanced Raman Scattering-Based Lateral Flow Assay Strips Using Highly Symmetric Gold Nanostars. ACS Appl. Nano Mater..

[49] Zhan L., Guo S.-Z., Song F., Gong Y., Xu F., Boulware D.R., McAlpine M.C., Chan W.C.W., Bischof J.C. (2017). The Role of Nanoparticle Design in Determining Analytical Performance of Lateral Flow Immunoassays. Nano Lett..

[50] Posthuma-Trumpie G.A., Korf J., van Amerongen A. (2008). Lateral flow (immuno)assay: Its strengths, weaknesses, opportunities and threats. A literature survey. Anal. Bioanal. Chem..

[51] Ayawei N., Ebelegi A.N., Wankasi D. (2017). Modelling and Interpretation of Adsorption Isotherms. J. Chem..

[52] Fabris L. (2020). Gold Nanostars in Biology and Medicine: Understanding Physicochemical Properties to Broaden Applicability. J. Phys. Chem. C.

[53] Jain P.K., Lee K.S., El-Sayed I.H., El-Sayed M.A. (2006). Calculated Absorption and Scattering Properties of Gold Nanoparticles of Different Size, Shape, and Composition: Applications in Biological Imaging and Biomedicine. J. Phys. Chem. B.

[54] Lu Z.-Y., Chan Y.-H. (2024). The importance of antibody orientation for enhancing sensitivity and selectivity in lateral flow immunoassays. Sensors Diagn..

