# Supplementary material for: Beyond Spheres: Evaluating Gold Nano-Flowers and Gold Nano-Stars for Enhanced Aflatoxin B1 Detection in Lateral Flow Immunoassays
Source: Biosensors (Basel). 2025 Aug 1;15(8):495. doi: 10.3390/bios15080495 (PMC12384835; doi:10.3390/bios15080495)
Supplement: Supplementary file 1 [file biosensors-15-00495-s001.zip › biosensors-3778899-supplementary.pdf]

Beyond Spheres: Evaluating gold nano flowers and gold nano stars for enhanced aflatoxin B1 detection in lateral flow immunoassays.

Vinayak Sharma[1,2 *], Bilal Javed[1], Hugh J. Byrne[2] & Furong Tian[1,2 *]

1 School of Food Science and Environmental Health, College of Sciences and Health Technological University Dublin, Dublin, Ireland

2 Nanolab Research Centre , Physical to Life Sciences Research Hub, Technological University Dublin, Dublin, Ireland

Supplementary Information

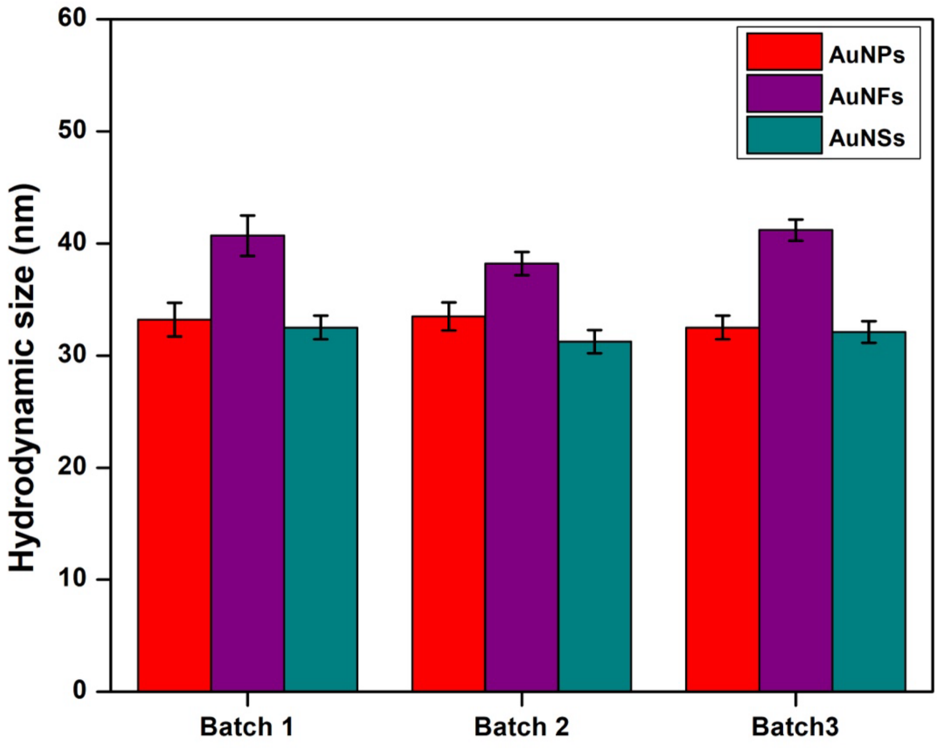

Supplementary Figure S1: Nano label synthesis batch 1 to batch 3 variability in hydrodynamic size (nm).

Supplementary Table S1:Mean size and CV(%) of synthesized nano labels across 3 batches.

	Mean Size (nm) ± SD	CV (%)
**AuNPs**	32.7 ± 0.8	2.4
**AuNFs**	39.7 ± 1.3	3.3
**AuNSs**	31.8 ± 0.5	1.6

To assess batch-to-batch consistency of nanoparticle synthesis, hydrodynamic diameters were measured across three independently synthesized batches for each nanoparticle morphology. The coefficient of variation (CV%) was found to be below **5%** in all cases (Table S1), confirming high reproducibility and stability of the synthesis protocol. All the measurements were taken in triplicate (n=3) for each of the three batches and the error bars represent the standard deviation. The final error margins are for all the batch and their mean size distribution.

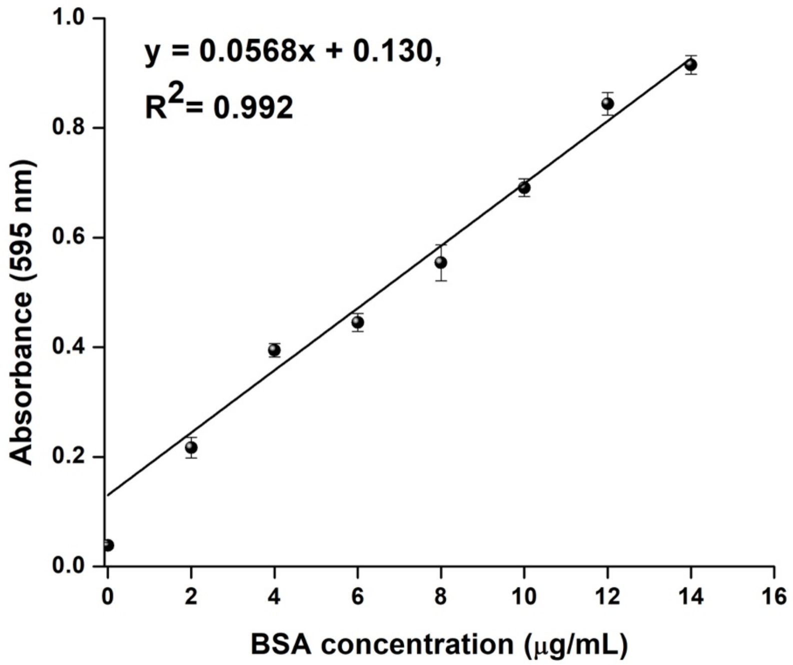

Supplementary Figure S2: Standard curve for BSA protein for the quantification of immobilization efficiency of nano labels.

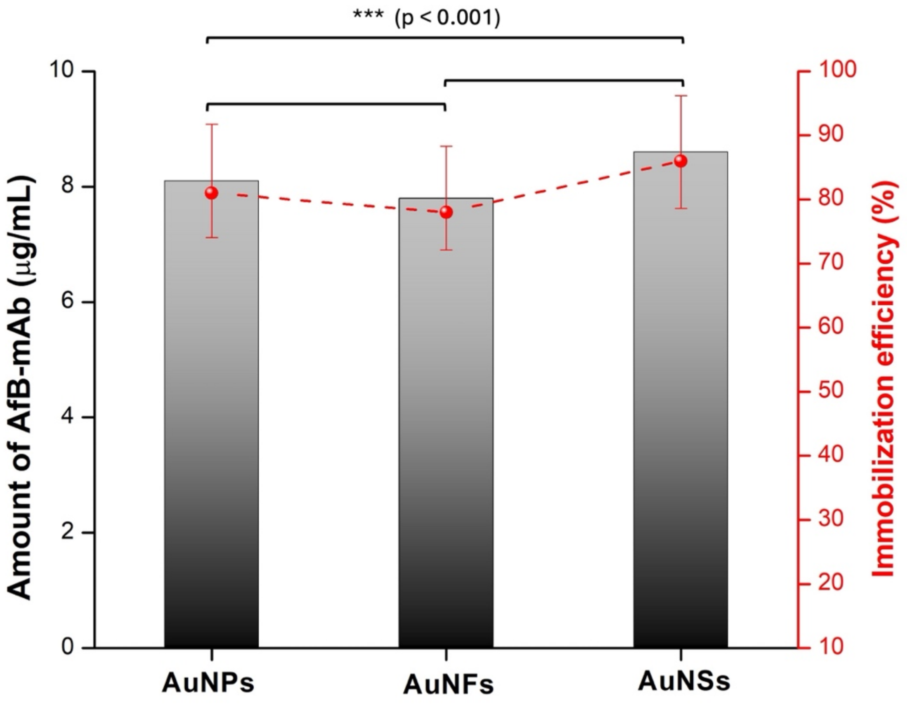

Supplementary Figure S3: Amount of anti-aflatoxin antibody immobilized and their immobilization efficiency calculated using Bradford assay**.**

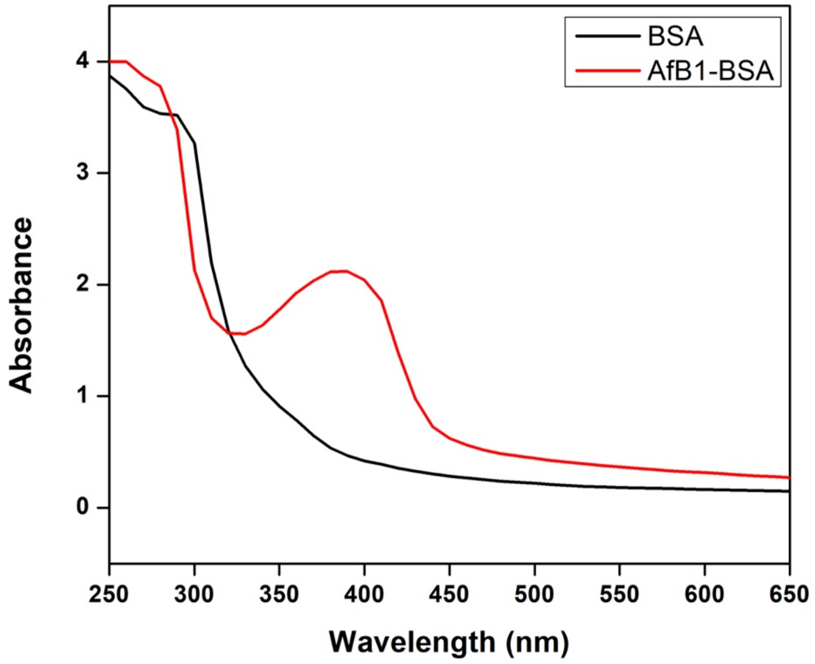

Supplementary Figure S4: UV-Vis spectroscopy of the synthesized AFB-BSA hapten as immunoreagent for test line.

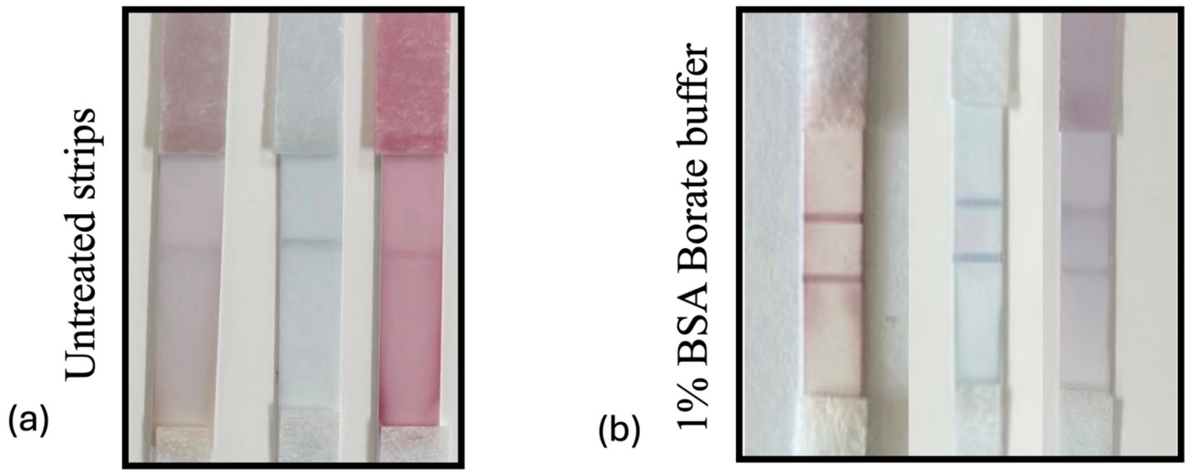

Supplementary Figure S5: LFIA images treated vs untreated nitrocellulose membrane.(a) untreated with BSA leading to deposition of the nano labels on the membrane; (b) membrane treated with 1% BSA in borate buffer.
